# Supplementary material for: An Assessment of the Effect of Progressive Water Absorption on the Interlaminar Strength of Unidirectional Carbon/Epoxy Composites Using Acoustic Emission
Source: Sensors (Basel). 2021 Jun 25;21(13):4351. doi: 10.3390/s21134351 (PMC8271434; doi:10.3390/s21134351)
Supplement: Supplementary file 1 [file sensors-21-04351-s001.zip › sensors-1257257-supplementary.pdf]

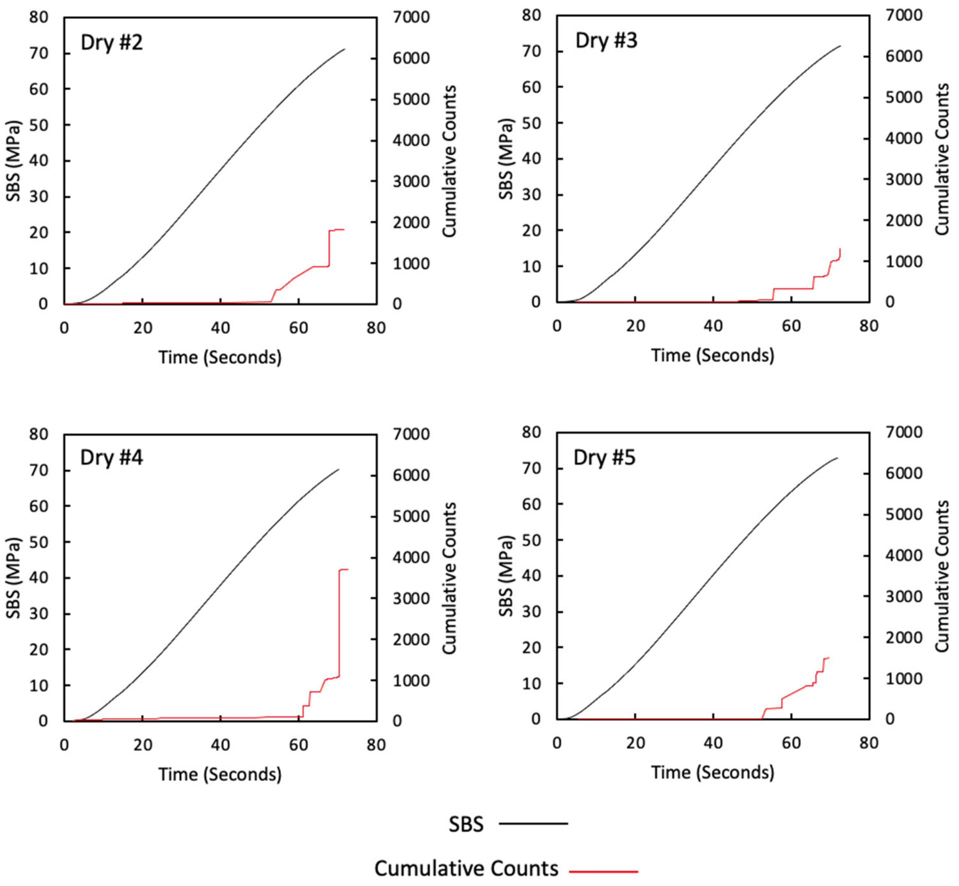

Figure 1: AE cumulative counts versus time for all remaining dry specimens.

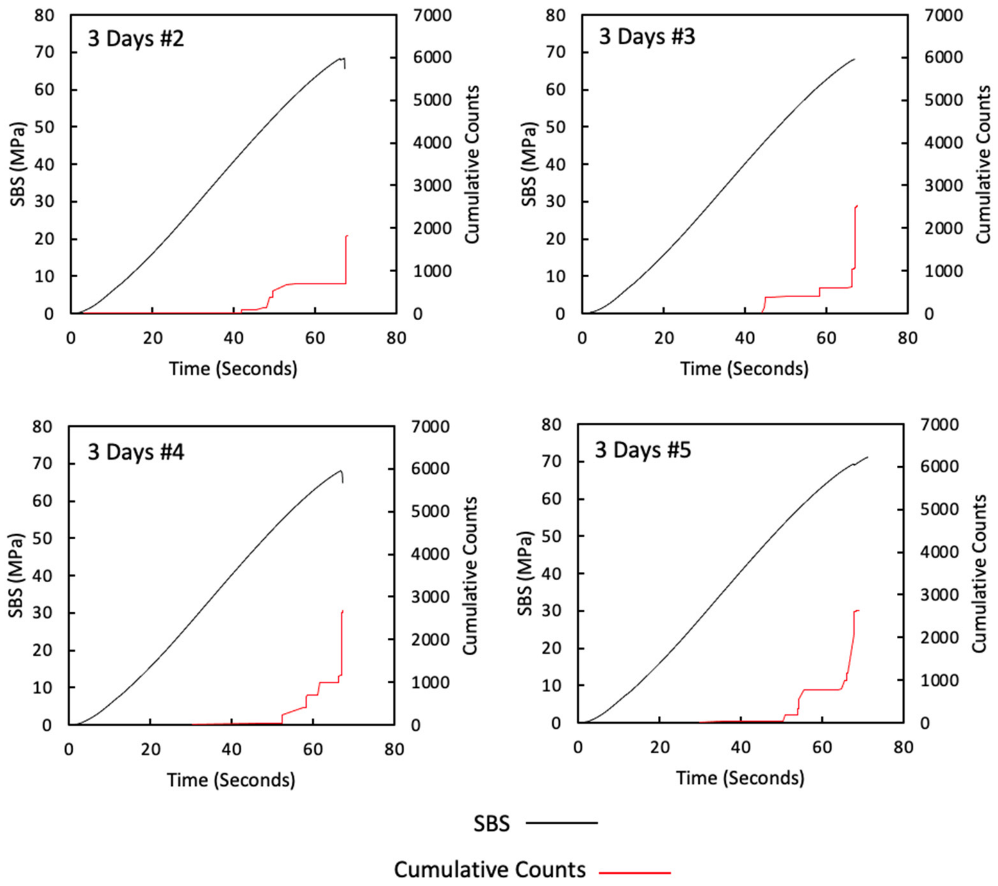

Figure 2: AE cumulative counts versus time for all remaining 3-day water immersed specimens.

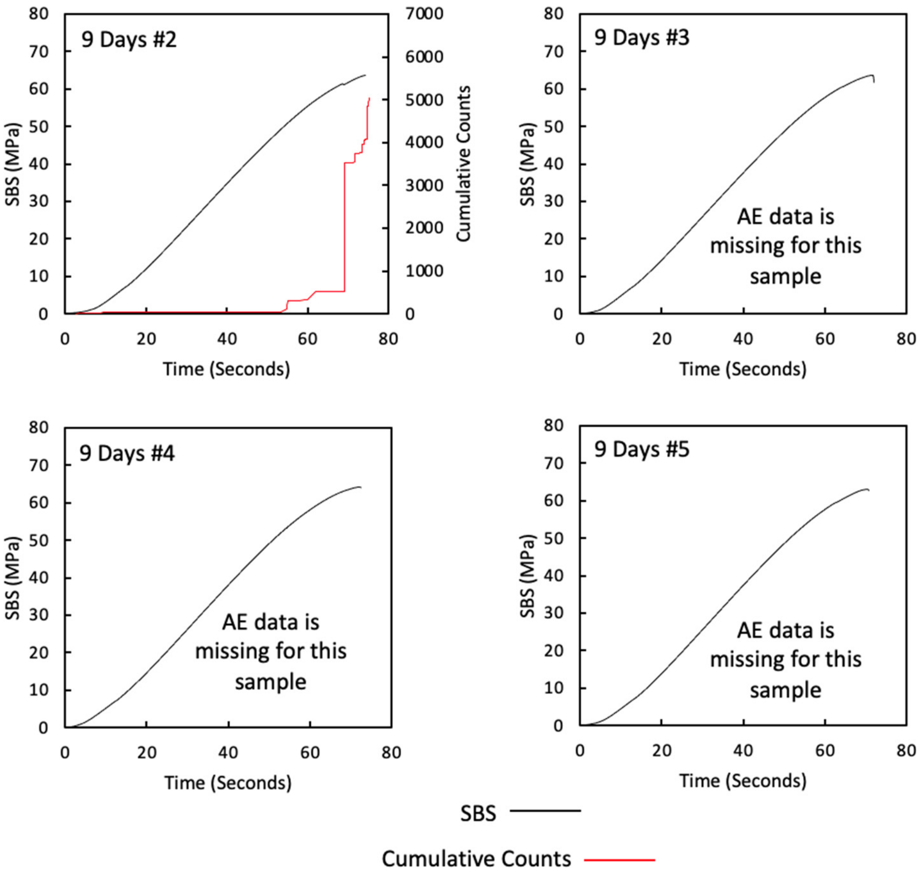

Figure 3: AE cumulative counts versus time for all remaining 9-day water immersed specimens.

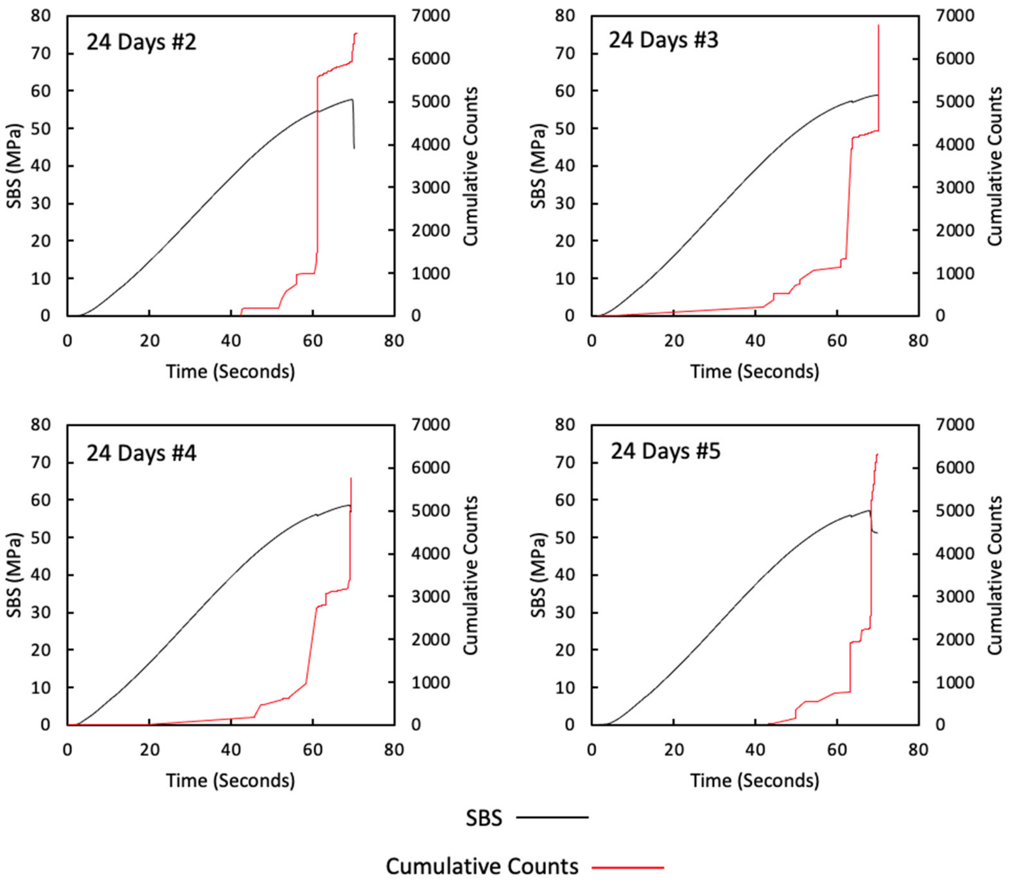

Figure 4: AE cumulative counts versus time for all remaining 24-day water immersed specimens.

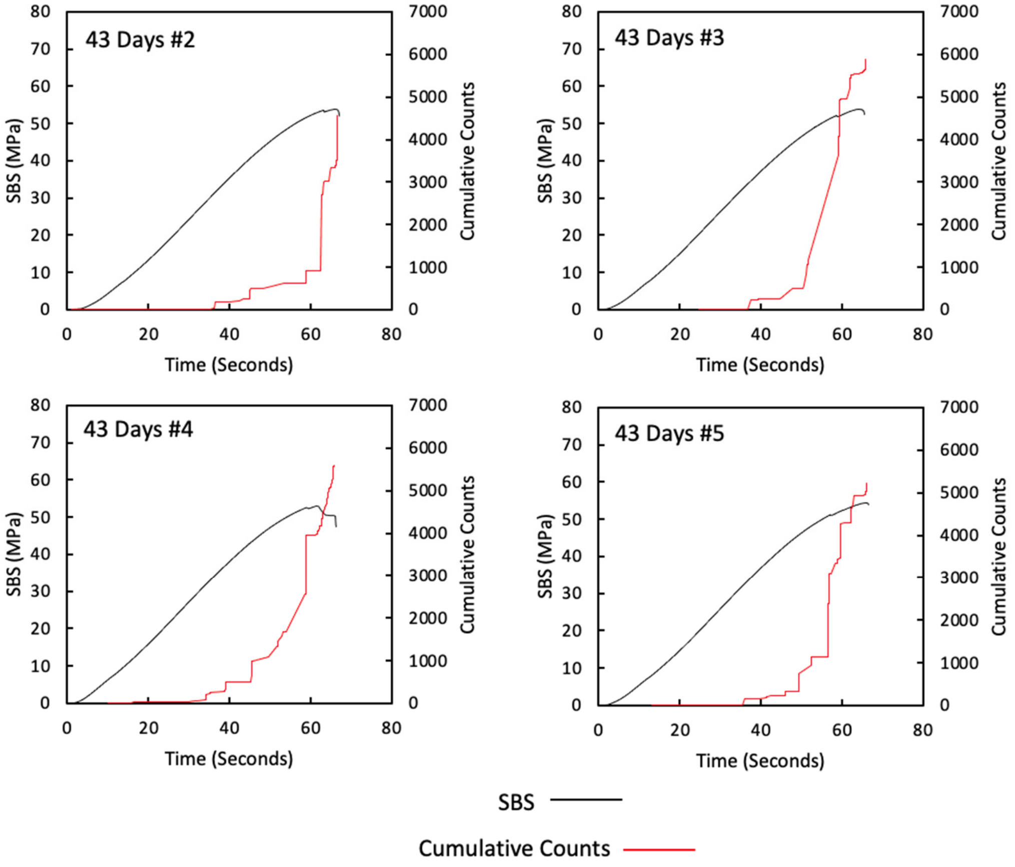

Figure 5: AE cumulative counts versus time for all remaining 43-day water immersed specimens.

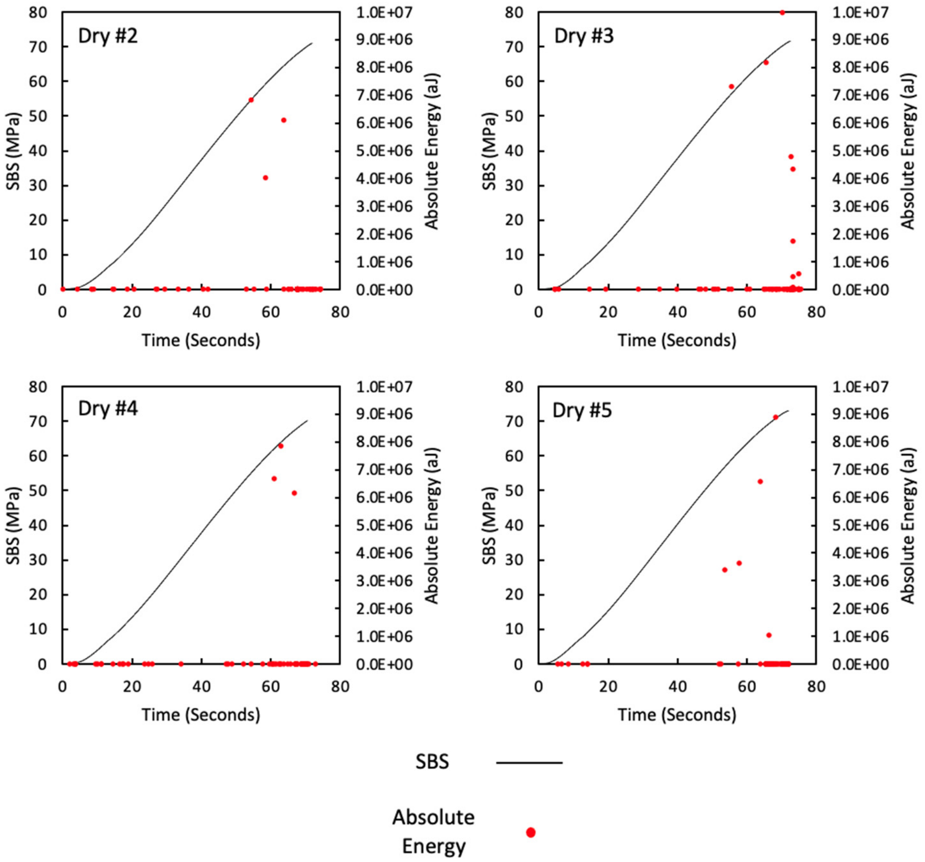

Figure 6: AE absolute energy versus time for all remaining dry specimens with absolute energy maximum scale bar of 1.0E+07.

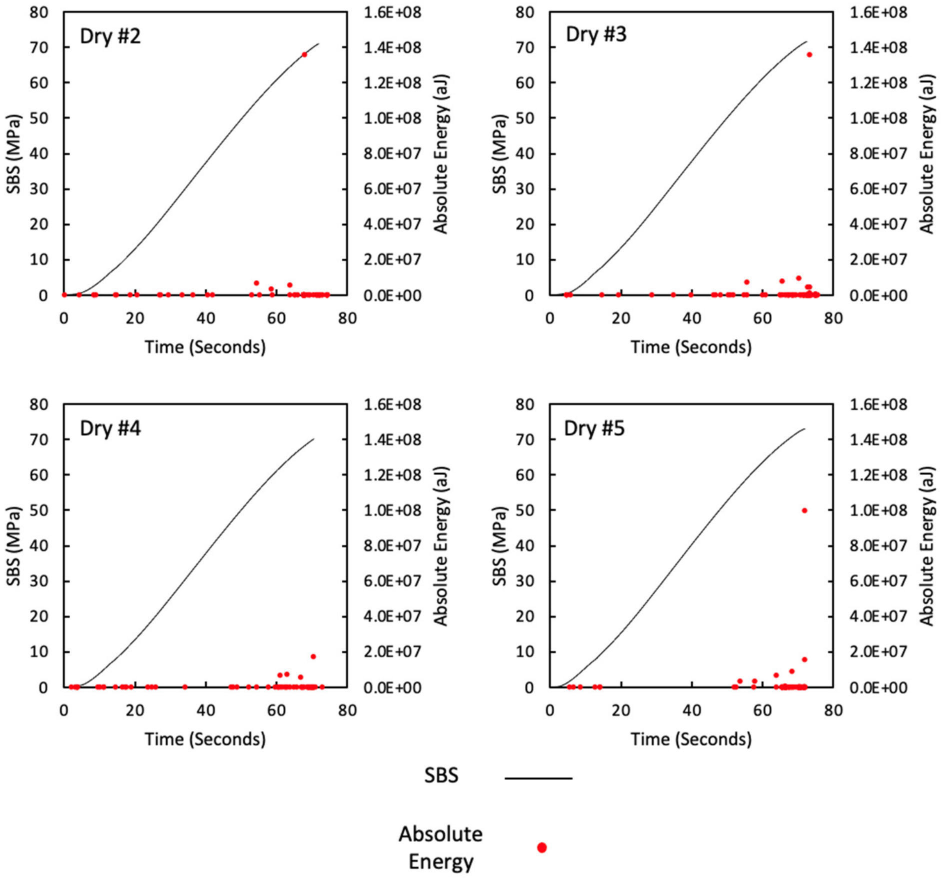

Figure 7: AE absolute energy versus time for all remaining dry specimens with absolute energy maximum scale bar of 1.6E+08.

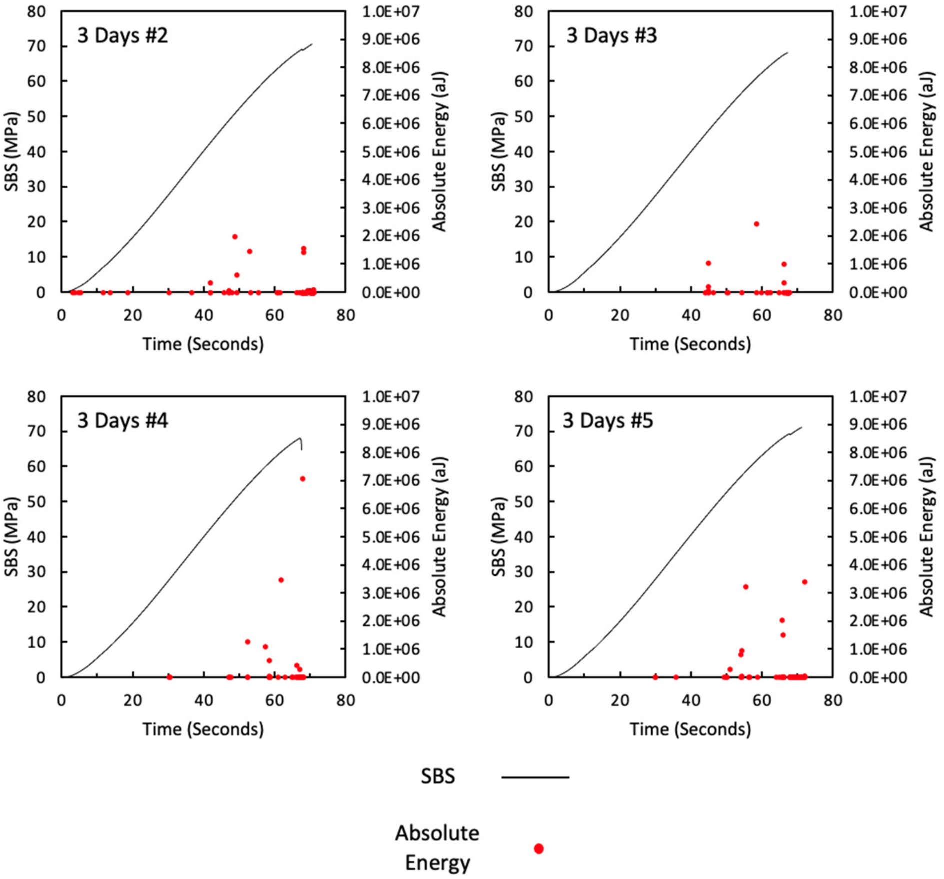

Figure 8: AE absolute energy versus time for all remaining 3-day water immersed specimens with absolute energy maximum scale bar of 1.0E+07.

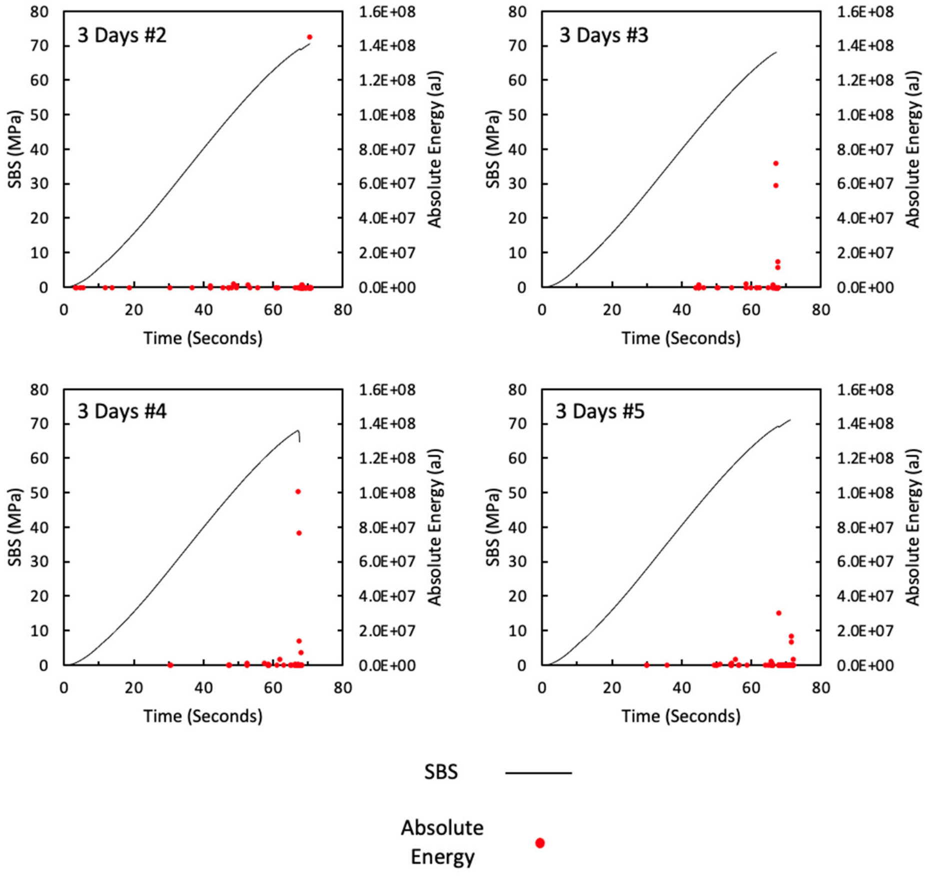

Figure 9: AE absolute energy versus time for all remaining 3-day water immersed specimens with absolute energy maximum scale bar of 1.6E+08.

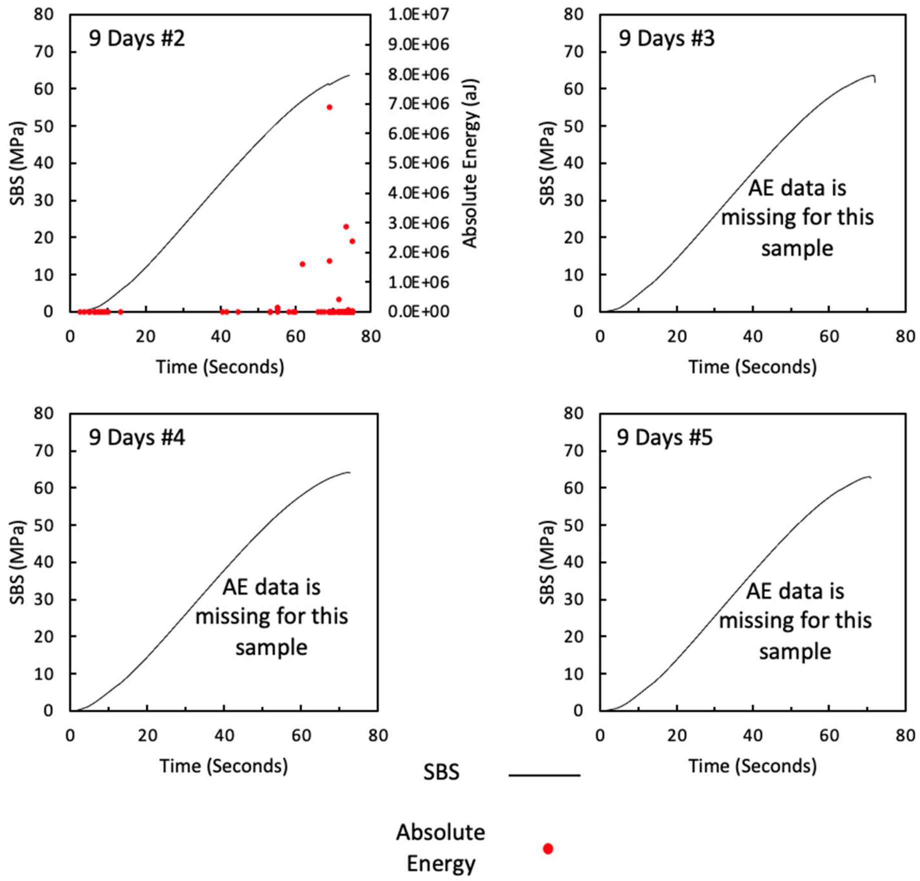

Figure 10: AE absolute energy versus time for all remaining 9-day water immersed specimens with absolute energy maximum scale bar of 1.0E+07.

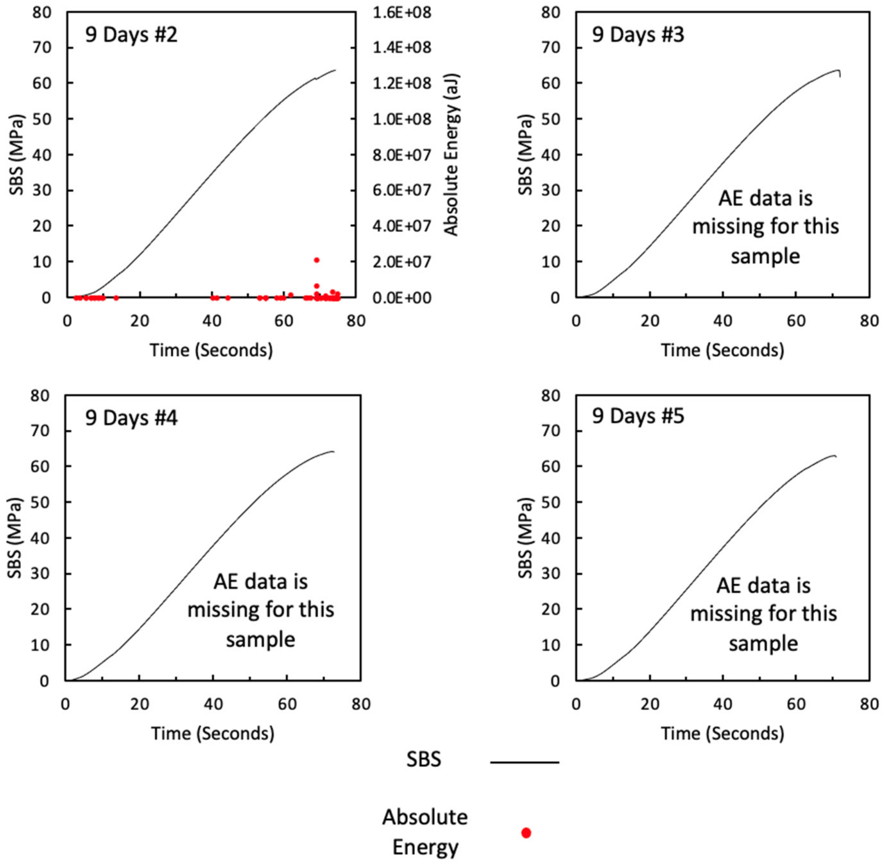

Figure 11: AE absolute energy versus time for all remaining 9-day water immersed specimens with absolute energy maximum scale bar of 1.6E+08.

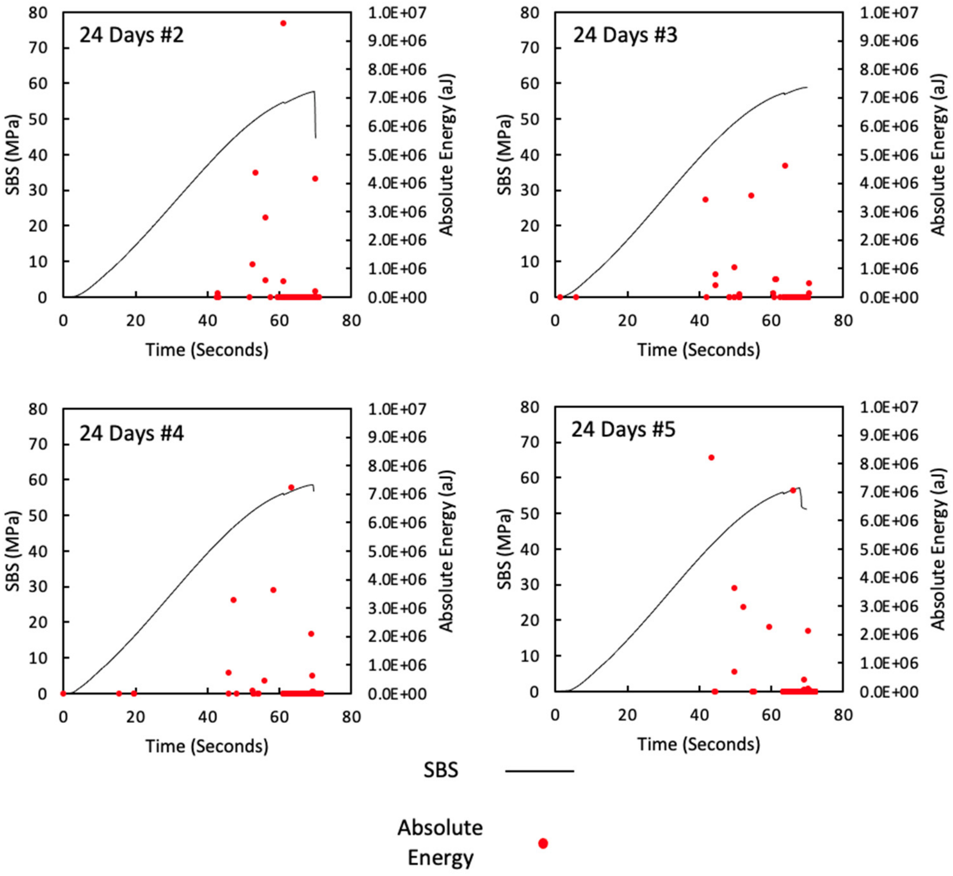

Figure 12: AE absolute energy versus time for all remaining 24-day water immersed specimens with absolute energy maximum scale bar of 1.0E+07.

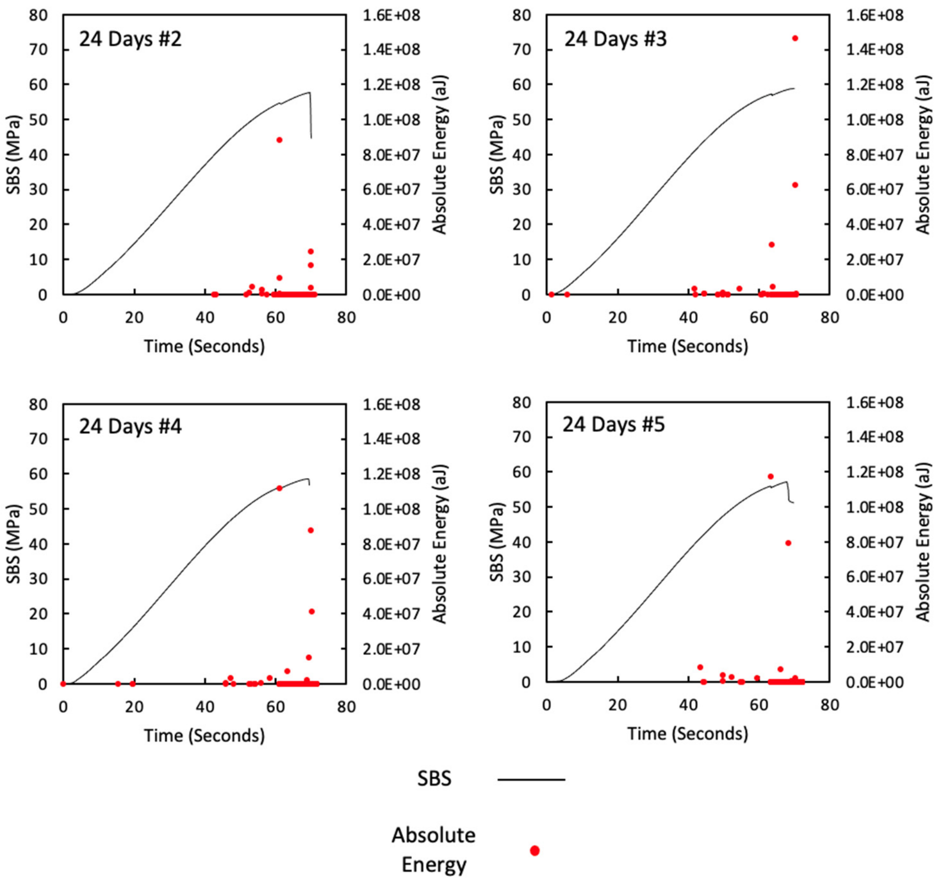

Figure 13: AE absolute energy versus time for all remaining 24-day water immersed specimens with absolute energy maximum scale bar of 1.6E+08.

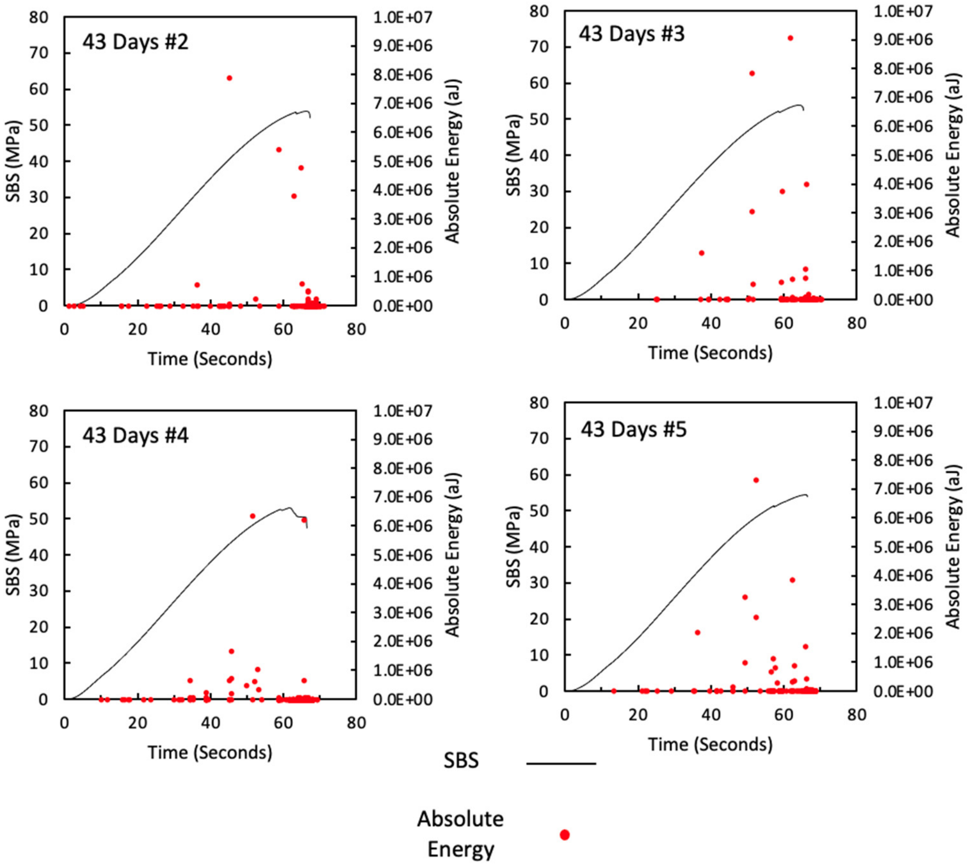

Figure 14: AE absolute energy versus time for all remaining 43-day water immersed specimens with absolute energy maximum scale bar of 1.0E+07.

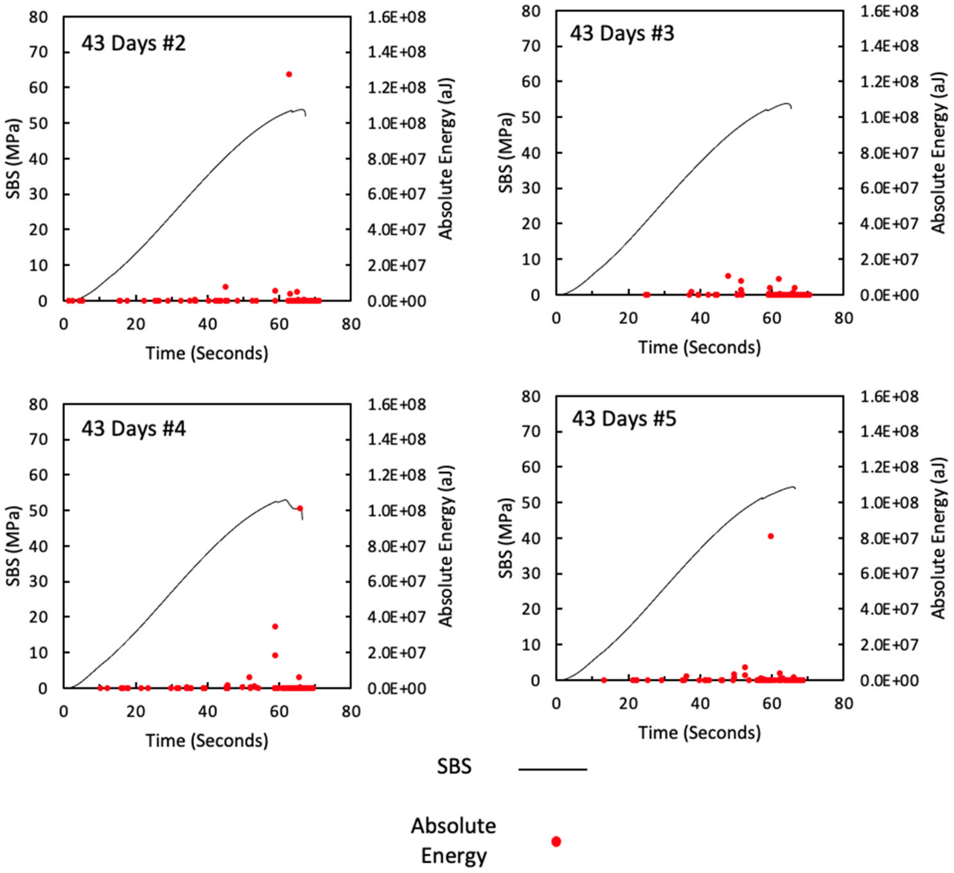

Figure 15: AE absolute energy versus time for all remaining 43-day water immersed specimens with absolute energy maximum scale bar of 1.6E+08.

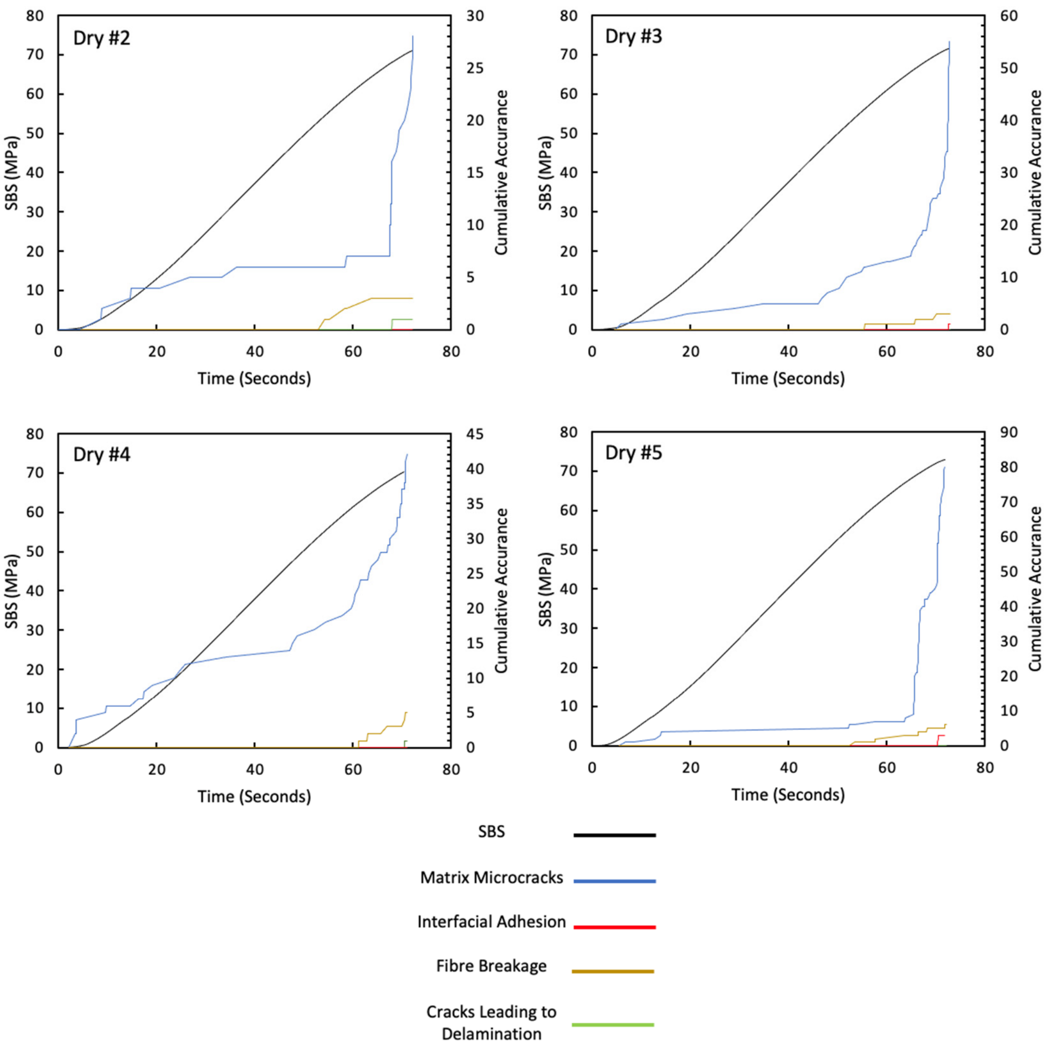

Figure 16: The representative damage mechanism based on the observed amplitude and duration data from the AE signals for all remaining dry specimens.

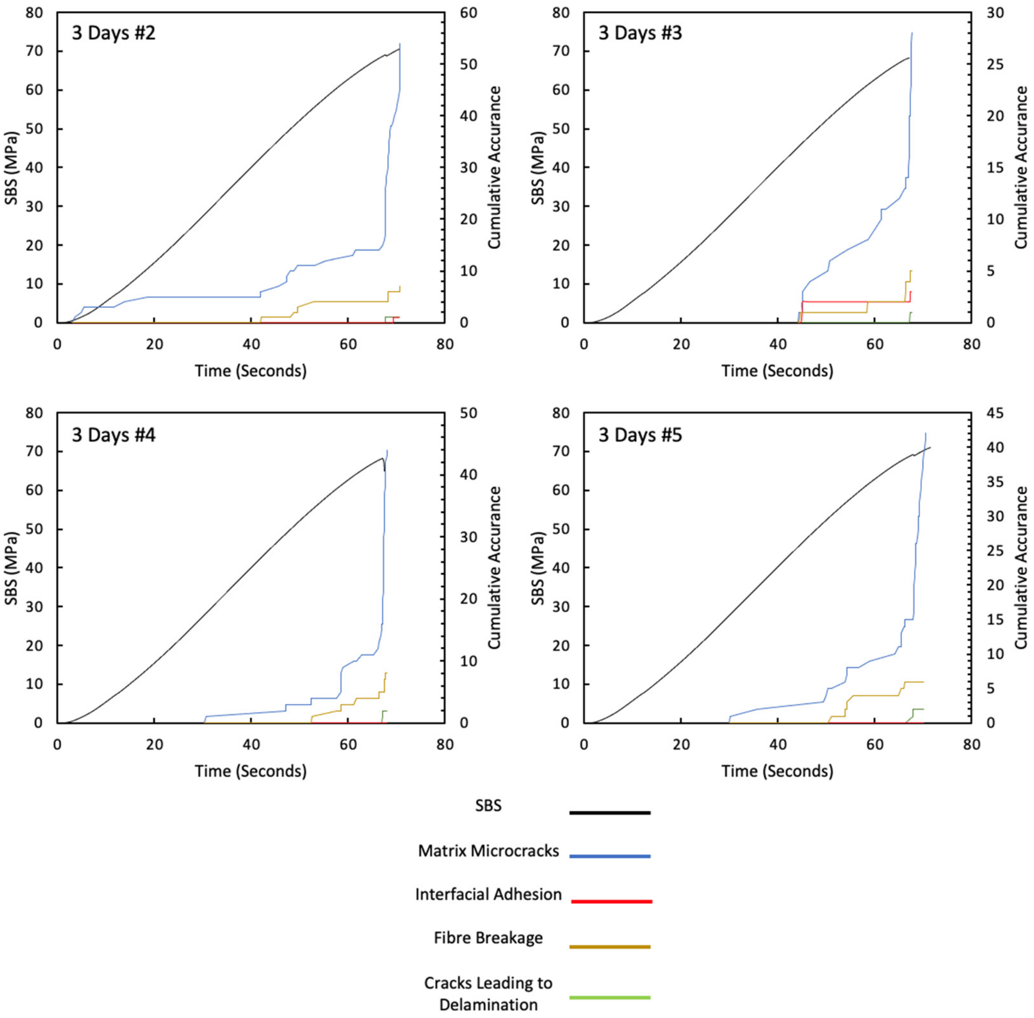

Figure 17: The representative damage mechanism based on the observed amplitude and duration data from the AE signals for all remaining 3-day water immersed specimens.

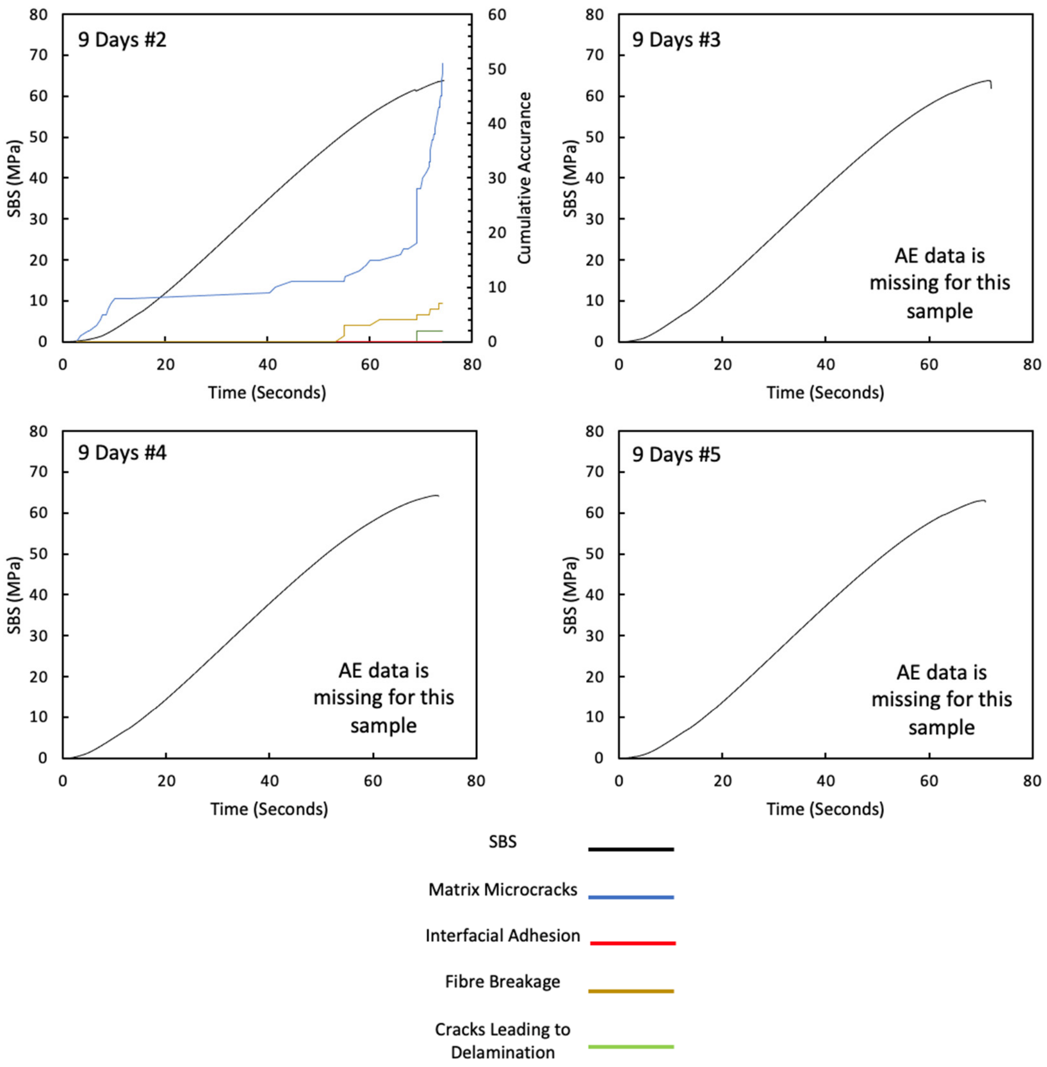

Figure 18: The representative damage mechanism based on the observed amplitude and duration data from the AE signals for all remaining 9-day water immersed specimens.

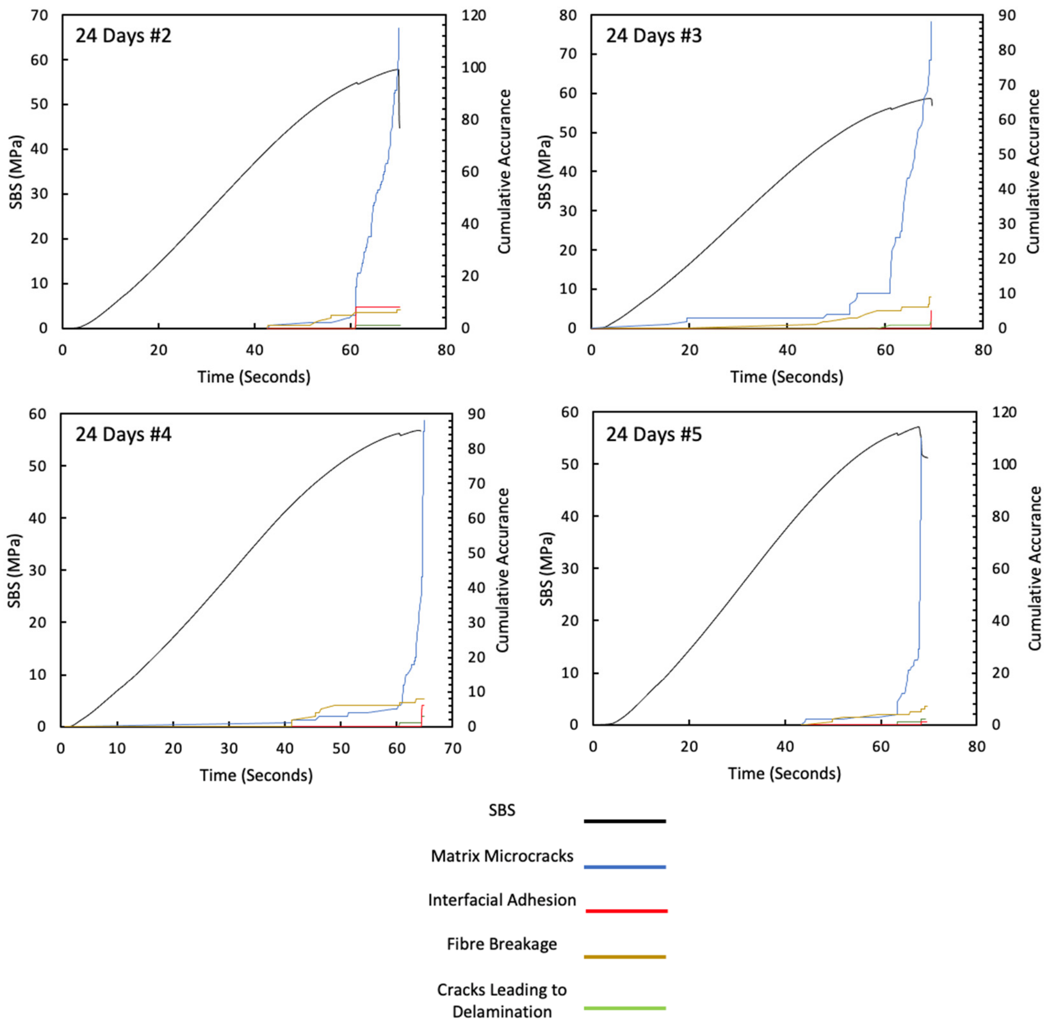

Figure 19: The representative damage mechanism based on the observed amplitude and duration data from the AE signals for all remaining 24-day water immersed specimens.

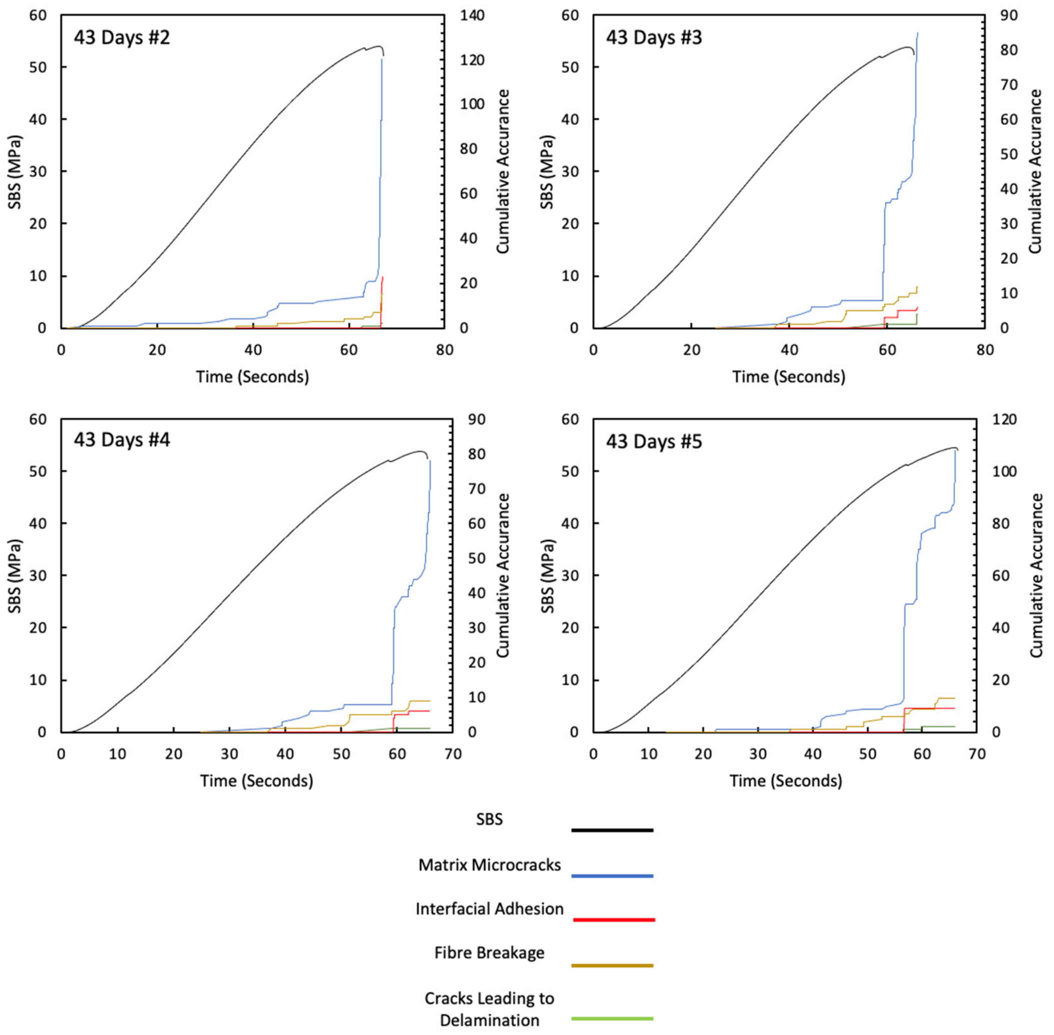

Figure 20: The representative damage mechanism based on the observed amplitude and duration data from the AE signals for all remaining 43-day water immersed specimens.
